# Small Colony Variants and Single Nucleotide Variations in Pf1 Region of PB1 Phage-Resistant *Pseudomonas aeruginosa*

**DOI:** 10.3389/fmicb.2016.00282

**Published:** 2016-03-09

**Authors:** Wee S. Lim, Kevin K. S. Phang, Andy H.-M. Tan, Sam F.-Y. Li, Dave S.-W. Ow

**Affiliations:** ^1^Agency for Science, Technology and Research, Bioprocessing Technology InstituteSingapore, Singapore; ^2^NUS Graduate School for Integrative Sciences and Engineering, National University of SingaporeSingapore, Singapore; ^3^NUS Environmental Research Institute, National University of SingaporeSingapore, Singapore; ^4^Lee Kong Chian School of Medicine, Nanyang Technological UniversitySingapore, Singapore; ^5^Department of Chemistry, Faculty of Science, National University of SingaporeSingapore, Singapore

**Keywords:** *Pseudomonas aeruginosa*, phage therapy, phage resistance, small colony variants, Pf1 region

## Abstract

Phage therapy involves the application of lytic bacteriophages for treatment of clinical infections but bacterial resistance may develop over time. Isolated from nosocomial infections, small colony variants (SCVs) are morphologically distinct, highly virulent bacterial strains that are resistant to conventional antibiotics. In this study, SCVs was derived from *Pseudomonas aeruginosa* exposed to the lytic bacteriophage PB1 and these cells were resistant to subsequent phage infection by PB1. To elucidate the mechanism of the SCV phage resistance, we performed phenotypic assays, DNA microarrays and whole-genome sequencing. Compared with wild-type *P. aeruginosa*, the SCV isolate showed impaired biofilm formation, decreased twitching motility, reduced elastase and pyocyanin production. The SCV is also more susceptible to the antibiotic ciprofloxacin and exhibited higher syrface hydrophobicity than the wild-type, indicative of changes to cell surface lipopolysaccharide (LPS) composition. Consistent with these results, transcriptomic studies of SCV revealed up-regulation of genes involved in O-specific antigen (OSA) biosynthesis, suggesting the regulation of surface moieties may account for phage resistance. Western blot analysis showed a difference in OSA distribution between the two strains. Simultaneously, genes involved in aromatic and branched chain amino acid catabolism were down-regulated. Whole genome sequencing of the SCV revealed multiple single nucleotide variations within the Pf1 prophage region, a genetic locus known to play a crucial role in biofilm formation and to provide survival advantage via gene transfer to a subpopulation of cells. Insights into phenotypic and genetic changes in SCV gained here should help direct future studies to elucidate mechanisms underpinning phage resistance, leading to novel counter resistance measures.

## Introduction

The emergence of multidrug-resistant pathogens and the difficulties in developing new antibiotics have spurred the resurgence of interest in phage therapy, involving the use of lytic bacteriophages against specific pathogens as a form of treatment (DiMasi et al., 2003; Gilbert et al., 2003; Spellberg et al., 2004; Talbot et al., 2006; Keen, 2012; Ly-Chatain, 2014). *Pseudomonas aeruginosa* is an opportunistic pathogen found ubiquitously in urban and natural environments, e.g., soil, rivers and sewage. In hospital settings, bacteria colonizes the surfaces of medical equipment such as catheters, inhalers, nebulizers and tubings (Stamm, 1978; Kirschke et al., 2003), where they account for approximately 10% of nosocomial infections (System, 2004). *P. aeruginosa* have been isolated from urinary tract and inner ear infections, burn wounds and from the surface of human epithelia in cystic fibrosis patients (Govan and Deretic, 1996; Lyczak et al., 2000; Lang et al., 2004; Taneja et al., 2004). Eradicating *P. aeruginosa* is not trivial as it has evolved various resistance mechanisms against conventional antibiotic therapies (Yoshimura and Nikaido, 1982; Nickel et al., 1985; Poole, 2004). Phage therapy has thus gained increasing consideration as an alternative treatment for antibiotic-resistant bacteria. Currently, phage therapies against methicillin-resistant *Staphylococcus aureus* and pathogenic *Escherichia coli* are in clinical trials (Harper and Enright, 2011). Studies have been carried out to elucidate how *P. aeruginosa* affects animal models of gut sepsis (Watanabe et al., 2007), burn wound (McVay et al., 2007) and lung infection (Morello et al., 2011). In one human clinical trial, Wright et al. (2009) administered a bacteriophage cocktail to treat chronic otitis. In another, Khawaldeh et al. (2011) reported the use of a lytic bacteriophage cocktail to treat a human patient suffering from *P. aeruginosa* urinary tract infection. Although these reports indicate that while phage therapy can be initially effective against *P. aeruginosa*, spontaneous phage resistance often occurs afterward, rendering the phage therapy ineffective.

Small colony variants (SCVs) of infectious strains of bacteria were first identified in 1910 (Proctor et al., 2006), so named because the colonies formed by SCVs are one 10th the colony size of their wild-type counterparts. SCVs exhibit higher antibiotic resistance than wild-type bacteria and they often form after exposure to high concentrations of antimicrobial agents such as gentamicin (Wei et al., 2011). Studies have also showed that SCVs could be induced in planktonic cultures of *P. aeruginosa* in response to infection by the lysogenic filamentous phage Pf4 (Webb et al., 2004; Hui et al., 2014). In the current study, a phage resistant SCV (F1 strain) of *P. aeruginosa* PAO1 strain (F0 strain) was successfully isolated using the lytic phage PB1. The first PB1 phage was first described in Holloway et al. (1960). Subsequently, a family of at least 42 other PB1-like bacteriophages against *P. aeruginosa* was discovered (Krylov et al., 1993; Pleteneva et al., 2008; Ceyssens et al., 2009). PB1 and PB1-like bacteriophages belong to the *Myoviridae* phage family and use bacterial lipopolysaccharide (LPS) as their receptor (Kropinski et al., 1977), and these lytic bacteriophages are a family of promising agents for phage therapy(Garbe et al., 2010; Krylov et al., 2013). Phage cocktail containing PB1-like *myoviridae* phages are currently use in clinical trials (Kwan et al., 2006; Merabishvili et al., 2009). The selection pressure imposed by PB1 phage allowed the isolation of SCVs which produce smaller colonies than their wild-type counterparts on agar plates. Besides determining the SCVs’ resistance to subsequent PB1 infections other characteristics such as their surface hydrophobicity, pyocyanin production, biofilm formation and cell lengths using microscopy were determined as well. The gene expression profiles of both wild-type and SCV *P. aeruginosa* were studied using DNA microarrays, and several pathways that could potentially confer phage resistance in SCV were identified. Whole genome sequencing enabled identification of point mutations and single nucleotide polymorphisms in the genome of SCVs that could have conferred a survival advantage and resulted in other phenotype changes in the SCVs.

## Materials and Methods

### Bacterial Strains and F1 Strain Isolation

*Pseudomonas aeruginosa* strain PAO1 (ATCC 47085) was designated as the wild-type F0 strain in this work. Glycerol stock of F0 was streaked on LB agar plates supplemented with 10 μg/mL tetracycline and incubated overnight at 37°C. For sub-culturing, 1 mL of overnight culture was added to 25 mL of LB broth diluted with 25 mL of reduced strength LB (20%) broth and incubated at 37°C, 225 rpm for all experiments unless otherwise stated. For phage infection, 500 μL of PB1 phage stock (1 × 10^10^ PFU/mL) was added to the subculture after allowing the subculture to recover at 37°C, 225 rpm for 1 h. Infected cultures were cultured for 24 h at 37°C, 225 rpm. The culture was streaked on fresh LB plates with 10 μg/mL tetracycline and incubated overnight at 37°C. The SCV was isolated (F1) for subsequent experiments.

### Determination of the Stability of SCV Phenotype

Single colonies of F0 and F1 were inoculated in 5 mL LB media and incubated at 37°C, 225 rpm for 6 h. The cultures were streaked onto agar plates and incubated at 37°C overnight. The colony size of both F0 and F1 were compared the following day. The SCV phenotype was determined to be stable as long as the colony size of F1 remained smaller than that of F0. The process was repeated for seven passages.

### OD600 Measurements, Cell Viability Assays, Generation Time Determination and Gram Staining and Microscopy

OD reading was measured at 600 nm using a UV-vis spectrophotometer in a 1 cm cuvette. Serial dilutions (10^-1^ to 10^-7^) of cultures were performed in 0.01% peptone. 100 μL of 10^-3^ to 10^-7^ dilutions with duplicates were spread on LB plates with 10 μg/mL tetracycline. Plates were incubated overnight at 37°C. Plates with colonies ranging from 25 to 300 were counted and the numbers obtained averaged. Morphologies on agar plates were observed. For the determination of generation time, hourly OD600 measurements were taken for 7 h and a growth curve was plotted (not shown). By measuring the gradient for the linear portion of the plot, the generation time could be determined. For microscopy studies, F0 and F1 cultures at mid-log were heat fixed and Gram-stained (Sigma) according to manufacturer’s protocol. The slides were viewed under a 100X objective with an Eclipse Ni-U microscope (Nikon) under oil immersion.

### Antibiotic Susceptibility Measurement with Etest

One mililiter of an overnight culture was diluted in 50 mL of LB media and incubated at 37°C, 225 rpm for 4 h. The turbidity of the culture was adjusted with 1X PBS to match that of MacFarland standard 0.5. Bacterial lawn of F0 or F1 was streaked onto Mueller-Hinton II agar plates with a cotton swab and plates allowed to dry for 5 min. An Etest strip (bioMérieux) containing gentamicin or ciprofloxacin was then placed onto the agar plate with sterile tweezers and the plates were incubated at 37°C overnight. The minimum inhibitory concentration (MIC) readings were read off at the point where the inhibition ellipse intersects the scale on the strip.

### Biofilm Formation Assay

The assay was adapted from the original protocol as described by Stepanović et al. (2000). A single colony of bacteria was inoculated in 5 mL Vogel-Bonner medium supplemented with 2% (w/v) glucose and incubated overnight at 37°C. Two hundred microliter of the overnight culture was seeded per well into a 96-well plate and the plate was further incubated for 24 h at 37°C. The culture was carefully aspirated with a pipette and each well was washed once with 200 μL of 1X sterile phosphate buffer saline (PBS), pH 7.5. The plate was inverted and dabbed dry onto paper towels after washing. One hundred and fifty microliter of methanol was added per well to fix the cells for 7 min and the methanol was then removed by removed by inverting the plate and gently flicking off any residual liquid. The plate was then dabbed dry onto paper towels. One hundred and fifty microliter of crystal violet was added per well to stain the attached cells for 30 min and the crystal violet was then removed by inverting and gently flicking the plate. The wells were then rinsed gently with tap water. The plate was inverted and finally dabbed dry onto paper towels. The amount of attached cells was then quantified via the solubilization of the bound crystal violet through the addition of 150 μL of 33% acetic acid into each well. The absorbance at 620 nm (A_620_) was measured using a plate reader.

### Elastase and Pyocyanin Assays

The assay was modified from the procedure given by Kamath et al. (1998) and Carlsson et al. (2011). Bacteria were grown for 60 h in King’s Medium A (King et al., 1954). Five mililiter of the overnight culture was centrifuged at 4600 rpm for 5 min at room temperature and the supernatant was collected and filtered through a 0.2 μm filter.

To measure the specific activity of elastase, 20 mg of Elastin-Congo Red (Sigma) substrate was added to 2 mL of reaction buffer (30 mM Tris-HCl; pH 7.5) in a 15 mL falcon tube. After warming the mixture to 37°C, 800 μL of filtered supernatant was added. The entire mixture was further incubated at 37°C, 225 rpm for 2 h. Elastin-Congo Red was pelleted by centrifugation at 4600 rpm for 10 min and the OD495 of the supernatant was measured with reaction buffer as the blank. The background absorbance was determined by measuring the absorbance of the reaction buffer and filtered supernatant without Elastin-Congo Red. Measurements were carried out in triplicate by a Infinite 200 PRO plate reader (Tecan) and the values were averaged and corrected for background absorbance. The elastase activity (OD495 hr^-1^ mL^-1^) of the filtered supernatant was calculated by adjusting the corrected OD495 values for incubation time, reaction volume and dilution factors. The amount of total protein in the filtered supernatant was determined with a Bradford assay, using the Coomassie Plus Assay Kit (Pierce) according to manufacturer’s instructions. Finally, the specific activity of elastase (OD495 mg protein^-1^ h^-1^) in the supernatant was determined by dividing the elastase activity with the total protein concentration.

For the measurement of pyocyanin concentration, 2 ml of chloroform was added to the filtered supernatant and vortexed for 30 s. The mixture was then centrifuged at 4600 rpm for 10 min to allow complete separation of the aqueous and organic phases. Eight hundred microliter of the blue chloroform phase was collected and mixed with 200 μL of 0.2 M HCl before vortexing for 30 s. One hundred fifty microliter of the pink layer was added to a 96 well plate and OD520 was measured with Infinite 200 PRO plate reader (Tecan). OD520 was multiplied by a factor of 17.072 to obtain the concentration of pyocyanin and the concentration of pyocyanin in the supernatant was determined by taking into account of dilution factors.

### Twitching Motility and Microbial Adhesion to Hydrocarbon (MATH) Assays

The twitching assay was carried out as described by Rashid and Kornberg (2000). Bacteria were stabbed onto twitch agar plates (1% agar LB plates). Twitch plates were incubated at 37°C for 48 h. The diameter of the zone of twitching was measured to estimation bacterial motility. To determine surface hydrophobicity, the MATH assay was adapted from the work of Pérez et al. (1998). Bacteria were first grown to mid-log in LB media. A 4 mL aliquot of the culture was centrifuged at 4600 rpm for 5 min. The supernatant was discarded and the pellet was re-suspended in 4 ml of PBS. This aliquot was then sub-divided into 2 aliquots of 2 ml each. Four hundred microliter of xylene was added to one of the tubes. Both tubes were vortexed for 2 min before allowing the tubes to stand for 30 min. Subsequently, 1 mL of aqueous phase from each aliquot was added to a cuvette and the OD OD600 measured (OD_0_ and OD_xylene_ representing the OD of the aliquot without and with xylene respectively). Surface hydrophobicity (H%) was calculated by the percentage change in turbidity before and after addition of xylene using the formula: $\frac{{OD}_{xylene}-{OD}_{o}}{{OD}_{o}}\times 100$

#### Statistical Analysis for Phenotypic Assays

The differences between F0 and F1 were assessed with the Mann–Whitney test (for cell length measurement as the distribution of cell length are not normally distributed) and the unpaired Student’s *t*-test (for all other assays) using GraphPad Prism. Values are reported as mean ± standard deviation (SD). *P*-values < 0.05 were considered statistically significant.

#### LPS Extraction and Western Analysis

The LPS extraction procedure was modified from that of Davis and Goldberg (2012). A 5 mL suspension of mid-log culture (OD 0.5) was centrifuged at 4600 rpm for 10 min and the supernatant was discarded. The bacterial pellet was resuspended in 200 μL 1X SDS buffer (2% β-mercaptoethanol, 2% SDS, 10% glycerol in 0.1M Tris-HCl pH 6.8, pinch of bromophenol blue) and boiled for 15 min. The solution was allowed to cool to room temperature for 15 min and 10 μL of 10 mg/mL Proteinase K was added. Samples were then incubated at 59oC overnight. Two hundred microliter of ice cold Tris-saturated phenol was added and samples were vortexed for 5 s. The mixture was incubated at 65oC for 15 min with occasional vortexing and then allowed to cool to room temperature. One mililiter of diethyl ether was added and vortexed for 5 s centrifuging at 20,600 *g* for 10 min. The bottom layer containing the LPS was carefully removed by a pipette. The remaining solution was re-extracted a second time by adding 200 μL of ice cold Tris-saturated phenol and following the steps above.

For LPS Western blot analysis, 15 μL of each sample was run on a denaturing 4–20% Tris-Glycine gel (Novex) according to manufacturer’s instructions. Monoclonal antibodies 5C7-4, 5c-101 and MF15-4 (MyBiosource) recognizing the inner, outer core and O-specific antigen (OSA) for O5 serotype were used as the primary antibody, and goat anti-mouse IgG-HRP (SantaCruz Biotechnology) was used as secondary antibody. Chemiluminescence detection was carried out using Clarity^TM^ Western ECL blotting substrate (Biorad), and the blot was visualized on ImageQuant LAS 500 (GE Healthcare Life Sciences).

### DNA Microarray

A total of four biological replicates of F0 and F1 each were used. Cells were grown to OD600 = 0.5 (mid-log) and 10 mL aliquots of culture were treated with 20 mL RNAProtect bacteria reagent (Qiagen) according to manufacturer’s protocol. RNA was isolated from these cells using RNAeasy MIDI kit (Qiagen). RNA was then converted to cDNA using Superscript II (Invitrogen). The cDNA was fragmented using DNAseI (New England Biolabs) and then labelled with Genechip DNA labeling reagent (Affymetrix) and terminal deoxynucleotidyl transferase (Promega). The hybridization cocktail was prepared using the GeneChip Hybridization, Wash and Stain Kit (Affymetrix) and hybridized to a *P. aeruginosa* genome array (Affymetrix). All steps performed were carried out according to the respective manufacturers’ protocol. Arrays were hybridized for 16 h at 50°C and then washed and stained with the GeneChip Hybridization, Wash and Stain Kit (Affymetrix). Data from the arrays were analyzed using Partek Genomics Suite. A list of differentially expressed genes (DEGs) was constructed using the criteria of *p* < 0.05 (one-way ANOVA) and a fold change (FC) of 1.5. Data are deposited in the Gene Expression Omnibus (GEO) database under accession number GSE75654.

### Next Generation Sequencing (NGS)

The Puregene kit (Qiagen) was used to isolate genomic DNA from overnight bacterial cultures, according to manufacturer’s instructions. The quality of the isolated gDNA was assessed through Nanodrop (Thermo Scientific). A total of five biological replicates for F1, four biological replicates for F0 were used. Genomic library preparation was carried out using Nextera XT kit (Illumina) and according to manufacturer’s instructions. All samples were pooled and ran on Mi-Seq (Illumina) in a 2 × 300 run. Raw reads were trimmed and aligned to *P. aeruginosa* PAO1 reference genome (Stover et al., 2000) using online tools^1^. The sequences were also compared with the annotated genome of *P. aeruginosa* strain PAO1^2^. Aligned reads were analyzed using Partek Genomic Suite to identify single nucleotide variations (SNVs). Only SNVs with a coverage >50, non-reference average base quality >20, and non-reference average mapping quality >20 were retained to generate an SNV list.

## Results

### SCV Phenotype of Derived Strain of F1

**FIGURE 1:**
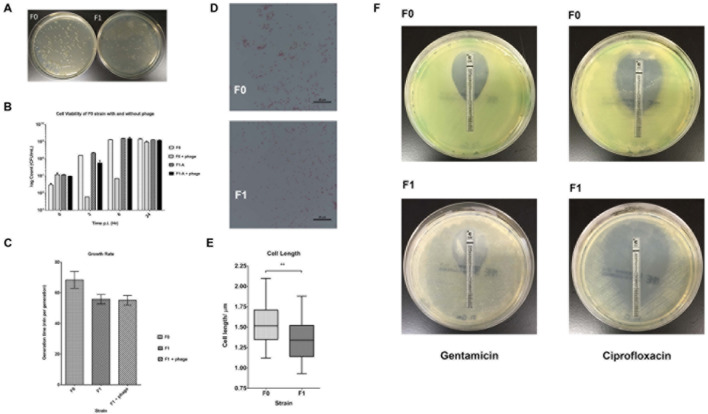
**Morphology and Cell Viability.** **(A)** Bacteria was plated on agar plates and incubated for 24 h at 37°C; *left- F0 (wt); right- F1 (SCV)*. **(B)** Bacteria count at 0, 3, 6, 24 h post infection (p.i.) for F0 and F1 strains. **(C)** Generation times (time taken for the doubling of population) for F0 and F1 strains, a total of three biological replicates were carried out. **(D)** Morphologies of gram-stained F0 and F1 strains, viewed under a 100x objective. **(E)** Distribution of cell length of 40 replicates each for F0 and F1 strains; Mann–Whitney test. F1 cells are significantly smaller than F0 cells. **(F)** Antibiotic susceptibility of F0 and F1 strains to gentamicin (left panels) and ciprofloxacin (right panels). ^∗^*p* < 0.05, ^∗∗^*p* < 0.005, ^∗∗∗^*p* < 0.0005.

Wild-type *P. aeruginosa* PAO1 (F0) was cultured and infected with PB1 phage. After overnight incubation at 37°C and centrifugation at 225 rpm, the culture was streaked on the agar plate and further incubated. A number of morphologically distinctive small colonies was observed (**Figure 1A**) and a SCV was isolated (F1) from the plate. To check if SCV phenotype of F1 was stable, the cells were sub-cultured and plated on agar plates. **Figure 1A** shows the colony morphology of F0 and F1 strains after 24 h incubation at 37°C. F1 colonies consistently showed the typical small and transparent colonies of an SCV after subculturing and this phenotype persists after seven passages. It was hypothesized that there may be variations within the genome of F1 forming a genomic imprint that allows the SCV phenotype to be maintained. Since F1 was isolated under the selection pressure imposed by PB1 phage, it is possible that F1 is resistant to subsequent bacteriophage treatment. Both F0 and F1 were re-infected with PB1 and their cell viabilities at 0, 3, 6, and 24 h post infection were measured, using their uninfected counterparts as controls (**Figure 1B**). When F0 is infected with phage, its viability decreased after 3 h, then increased at 6 h and eventually attained 10^9^ CFU/ mL after 24 h. In contrast, the viability of F1 increased steadily, in a manner similar to uninfected F0, to 10^9^ CFU/mL after 24 h, regardless of whether phage was added. The generation time for F1 strain as determined by OD600 measurement is lower than F0 (**Figure 1C**), which may suggest that F1 has a faster growth rate, contradicting previous reports on SCVs being slow-growing (Proctor et al., 2006). However, cell viability measurements showed there is no difference in growth (**Figure 1B**). This is because F0 and F1 cells have different sizes, thus producing the dichotomy between optical density and cell viability (Sutton, 2011). A representative set of 40 cells from each of F0 and F1 strains were picked for cell length measurement. As shown in **Figures 1D,E**, F1 has a distribution of cell lengths which is noticeably shorter than that of F0. From the Etest results, there appears to be no difference in gentamicin susceptibility but an increase in ciprofloxacin susceptibility in F1 (**Table 1**; **Figure 1F**).

**Table 1:** Antibiotic susceptibility measurement with Etest.

	Minimum inhibitory concentration (μg/mL)	
Antibiotic	F0	F1
Gentamicin	2.000	1.500
Ciprofloxacin	0.094	0.016


**FIGURE 2:**
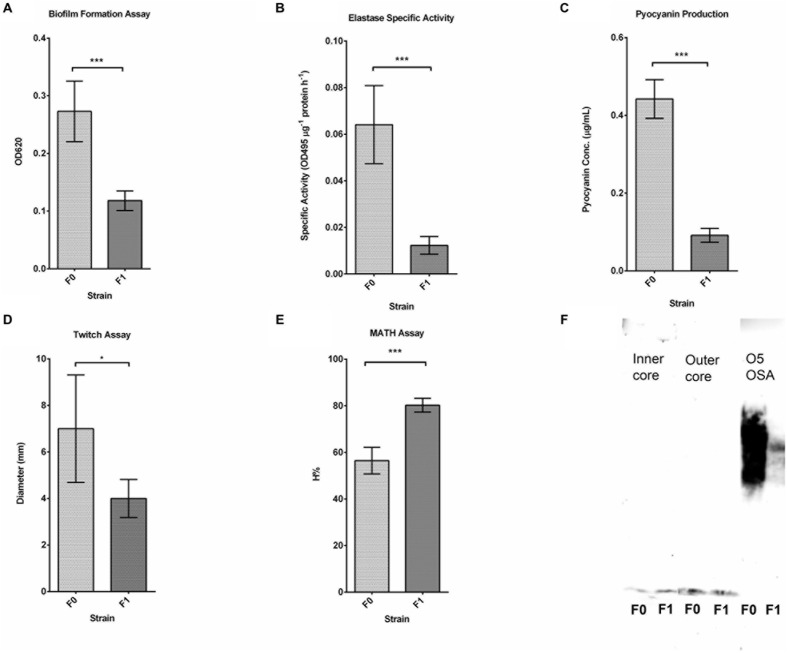
**Results for phenotypic assays.** **(A)** Biofilm formation assay of three replicates of F0 and F1 strains; Student’s *t*-test. F1 cells have a significantly lower OD260 than F0, indicating fewer attached cells since fewer of them are stained. **(B)** Elastase assay of five replicates of F0 and F1 strains; Student’s *t*-test. F1 cells produce significantly lower elastase specific activity than F0. **(C)** Pyocyanin production assay of five replicates of F0 and F1 strains, measured concurrently with elastase activity; Student’s *t*-test. F1 cells produce significantly lower amount of pyocyanin than F0. **(D)** Twitch assay for seven replicates of F0 and F1 strains; Student’s *t*-test. F1 cells have a significantly lower twitching ability than F0. **(E)** MATH assay of 12 replicates of F0 and F1 strains; Student’s *t*-test. F1 cells have a significantly higher H% than F0 cells. **(F)** Western blot analysis of LPS preparations from F0 and F1 strains, tested using antibodies for inner, outer core and OSA. ^∗^*p* < 0.05, ^∗∗^*p* < 0.005, ^∗∗∗^*p* < 0.0005.

Several phenotypic assays (biofilm formation, elastase/pyocyanin assays, twitching motility, microbial adhesion to hydrocarbon or MATH assay,) were also carried out to characterize the other physiological differences between F0 wild-type and F1 SCV strains. Using crystal violet to quantify biofilm on 96-wells (**Figure 2A**), it was found that biofilm formed by F1 cells on the surface of the wells have a lower absorbance at 620 nm than F0; indicating a lower amount of biofilm formation by the F1 compared with the F0 cells. The elastase and pyocyanin assays (**Figures 2B,C**) detected lower amounts of elastase and pyocyanin in the supernatant of F1 cultures compared to F0. Since both elastase and pyocyanin are virulence factors whose functions include inhibiting the growth of competing microflora, damaging pulmonary tissues and contributing to *P. aeruginosa* persistence in cystic fibrosis patients (Lau et al., 2004), decreased production of these factors by the phage-resistant F1 could result in lesser adverse effects if phages were used to treat patients. The twitching assay (**Figure 2D**) showed that the twitching zone of F1 is smaller than that of F1, indicating that F1 cells have lower surface-associated motility than F0 in 1% agar. The MATH assay is a measurement of surface hydrophobicity, based on the partitioning of cells between organic and aqueous phases. H% of F1 is higher than that of F0 (**Figure 2E**), suggesting that F1 may have a more hydrophobic cell surface, which may be attributed to an increase in surface LPS or changes to LPS structure. From our Western blot analysis, we observed a different banding pattern for OSA (formerly known as B-band) between F0 and F1 (**Figure 2F**). F0 have a uniform distribution of OSA of different lengths while F1 seems to possess predominantly OSA of medium length and low amounts of shorter length OSA while devoid of full length OSA.

### DNA Microarray

**FIGURE 3:**
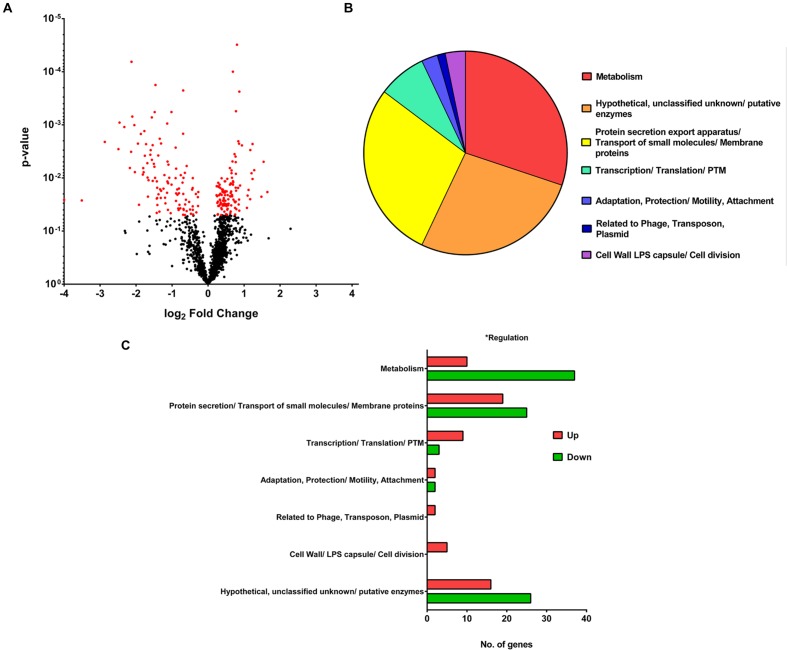
**Microarray analysis.** **(A)** Volcano Plot showing the fold change and *p*-value of the 1566 probesets. For a gene to be differentially expressed, it must satisfy the criteria of *p*-value <0.05 and fold change > 1.5 or < -1.5. One hundred and forty-eight differentially expressed genes (DEGs) are shown in red on the plot. **(B)** Pie chart showing PseudoCAP functional classification of DEGs, with metabolism, hypothetical proteins and protein secretion forming the majority. **(C)** Bar graph showing PseudoCAP functional classification of DEGs sorted according to their expression level.

To study the differences in gene expression between the wild-type F0 and SCV F1 strains, bacterial samples at mid-log growth phase were collected for RNA extraction and cDNA synthesis for DNA microarray transcriptome analysis. After array hybridization and scanning, data was normalized using the Gene Chip (GC) robust multi-array analysis (GCRMA) and probesets were filtered according to their signal intensity and standard deviation (Hackstadt and Hess, 2009). Probesets with low signal (<3.2), constituting noise, and with low standard deviation (<0.25) were removed, yielding 1566 probesets which met the threshold for analysis. This represented an average of 63% of the 5900 open reading frames (ORFs) detected on the array chip. Using a *p*-value criteria of <0.05 and a FC of 1.5 for a DEG, 148 DEGs (**Figures 3A,B**) were identified between F0 and F1. The 148 DEGs represented 2.5% of the total number of ORFs, of which 55 genes were upregulated while 93 were downregulated (**Supplementary Tables S1** and **S2**). Annotating these DEGs according to their PseudoCAP (*Pseudomonas* community annotation project) class functions (**Figure 3C**) revealed that the three largest groups of DEGs belong to metabolism (30%), protein secretion/transport (28%) and hypothetical proteins (27%).

**FIGURE 4:**
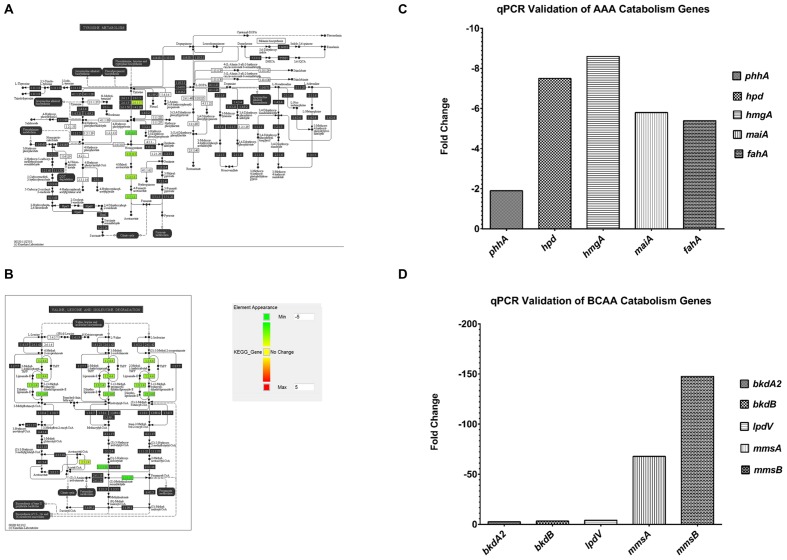
**Pathway analysis of microarray results.** **(A)** Pathway analysis- 5 genes, *phhA*, *hpd, hmgA, maiA* and *fahA* from the aromatic amino acid catabolism pathway were downregulated (green). **(B)** Pathway analysis- 5 genes, *bkdA2*, *bkdB, lpdV, mmsA* and *mmsB* from the branched chain amino acid catabolism pathway were downregulated (green). **(C)** A representative qPCR validation of *phhA*, *hpd, hmgA, maiA* and *fahA*, normalized to *rpoD*. **(D)** A representative qPCR validation of *bkdA2*, *bkdB, lpdV, mmsA* and *mmsB*, normalized to *rpoD*.

An interesting group of genes that were upregulated in F1 are the *P. aeruginosa* serotype O5 OSA biosynthesis cluster (**Table 2A**) – *wbpI, wbpH, wbpG, hisF2* and *hisF1*. OSA acts as the O-antigen for serotype O5 to which PAO1 belongs. This cluster contains 16 genes that are involved in the synthesis of LPS and three other genes that are not (Burrows et al., 1996). This group of genes is also found in serotypes O2, O16, O18, and O20 which have a structurally related O-antigen serogroup. Down-regulated genes include those involved in several amino acid catabolic pathways, such as aromatic amino acid catabolism (**Table 2B**) – *hpd, hmgA, maiA and fahA* and branched chain amino acid (BCAA) catabolism (**Table 2C**) -*bkdA2, bkdB, mmsA, mmsB* and *lpdV* and (**Figures 4A–D**). We proposed that the amino acids may be required as substrates for other pathways hence they are from being catabolized.

**Table 2A:** Up-regulated genes involved in *Pseudomonas aeruginosa* serotype O5 B-band LPS biosynthesis.

Locus	Gene	Function	Fold Change	*p*-value
PA3148	*wbpI*	Probable UDP-*N*-acetylglucosamine 2-epimerase	1.8	4.E-02
PA3149	*wbpH*	Probable glycosyltransferase WbpH	2.2	3.E-02
PA3150	*wbpG*	LPS biosynthesis protein WbpG	2.1	2.E-02
PA3151	*hisF2*	Imidazoleglycerol-phosphate synthase, cyclase Subunit	2.4	1.E-02
PA3152	*hisH2*	Glutamine amidotransferase	2.3	3.E-03


**Table 2B:** Down-regulated genes involved in aromatic amino acid catabolism.

Locus	Gene	Function	Fold Change	*p*-value
PA0865	*hpd^∗^*	4-hydroxyphenylpyruvate dioxygenase	-4.1	8.E-03
PA0870	*phhC*	Aromatic amino acid aminotransferase	-2.2	1.E-02
PA2007	*maiA^∗^*	Maleylacetoacetate isomerase	-2.7	3.E-02
PA2008	*fahA^∗^*	Fumarylacetoacetase	-3.2	5.E-03
PA2009	*hmgA^∗^*	Homogentisate 1,2-dioxygenase	-2.7	2.E-02


**Table 2C:** Down-regulated genes involved in branched chain amino acid catabolism.

Locus	Gene	Function	Fold Change	*p*-value
PA0744	–	Probable enoyl-CoA hydratase/isomerase	-4.1	1.E-03
PA0745	–	Probable enoyl-CoA hydratase/isomerase	-5.0	1.E-03
PA0747	–	Probable aldehyde dehydrogenase	-1.6	4.E-02
PA1984	*–*	NAD + dependent aldehyde dehydrogenase	-5.5	9.E-04
PA2000	*–*	Dehydrocarnitine CoA transferase, subunit B	-1.6	5.E-02
PA2001	*atoB*	Acetyl-CoA acetyltransferase	-1.9	1.E-02
PA2012	*gnyA*	Alpha subunit of geranoyl-CoA carboxylase	-2.6	4.E-02
PA2013	*gnyH*	Gamma-carboxygeranoyl-CoA hydratase	-2.6	2.E-02
PA2014	*gnyB*	Beta subunit of geranoyl-CoA carboxylase	-2.3	2.E-02
PA2247	*bkdA1*	2-oxoisovalerate dehydrogenase (alpha subunit)	-2.7	1.E-02
PA2248	*bkdA2^∗^*	2-oxoisovalerate dehydrogenase (beta subunit)	-3.4	4.E-03
PA2249	*bkdB^∗^*	Branched-chain alpha-keto acid dehydrogenase (lipoamide component)	-3.4	1.E-03
PA2250	*lpdV^∗^*	Lipoamide dehydrogenase-Val	-3.0	4.E-03
PA2553	*–*	Probable acyl-CoA thiolase	-5.6	3.E-03
PA2554	*–*	Probable short-chain dehydrogenase	-2.2	1.E-02
PA3569	*mmsB^∗^*	3-hydroxyisobutyrate dehydrogenase	-4.3	7.E-04
PA3570	*mmsA^∗^*	Methylmalonate-semialdehyde dehydrogenase	-4.5	6.E-03


*^∗^Denotes genes that were validated with qPCR.*

### Identifying Unique F1 Single Nucleotide Variations by Whole Genome Sequencing

**FIGURE 5:**
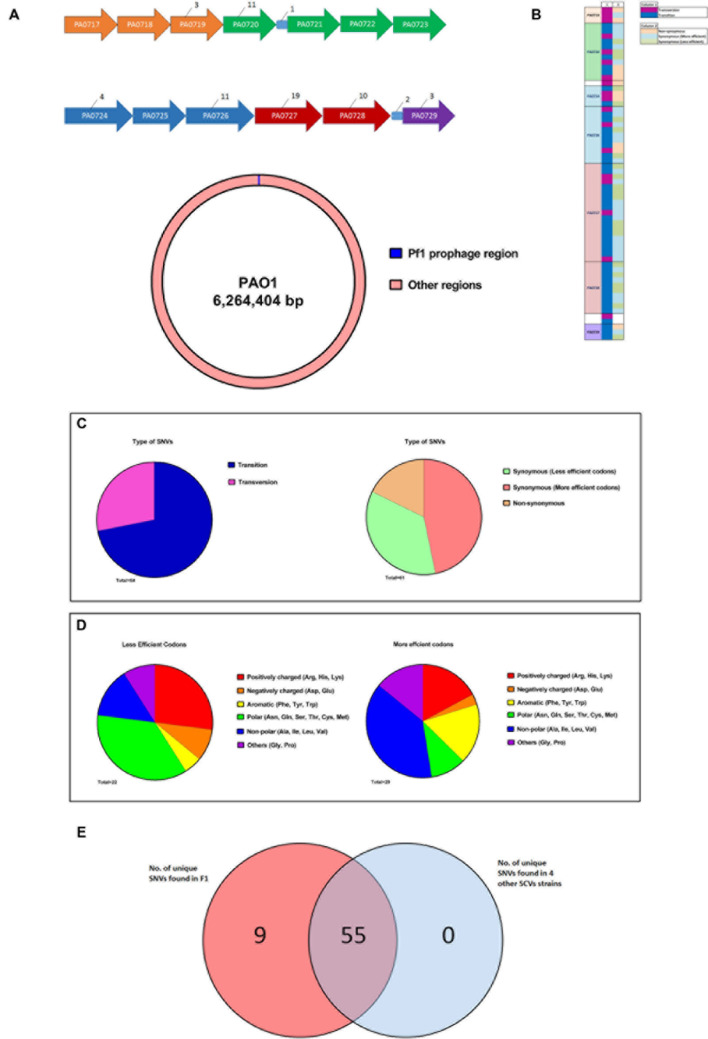
**Whole genome sequencing of F1 to identify unique SNVs.** **(A)** Figure showing the distribution of unique F1 strains SNV across the Pf1 prophage regions. All intergenic regions except those containing SNVs were removed for clarity. Numerical values indicate the number of SNVs found in each gene. Genes of the same color belongs to the same operon. **(B)** A schematic showing the size of Pf1 prophage region (∼8 kb) in relation the size of the PAO1 genome (∼6 Mb) **(C)** Synoymous SNVs were split into two groups based on whether a less or more efficient codon for a particular amino acid was used in F1 and the type of amino acid side chains were shown in the pie charts. Aromatic and branched chain amino acids (yellow and blue regions respectively) were preferentially using a more efficient codon. **(D)** Diagram showing the attributes of the SNVs found in each locus. **(E)** Venn diagram showing the distribution of these 64 SNVs across 4 other SNV isolated from F0. 55 of these SNVs are common to all five strains.

Since the SCV phenotype of F1 is stable, whole genome sequencing to identify single nucleotide variations (SNVs) was performed to elucidate the underlying genomic changes in F1 compared to wild-type F0. From the SNV list, we needed to identify SNVs that were present only in F1 but absent in F0. We pooled all the SNVs found in F1 and F0 separately and compared the SNVs. Using this approach, 64 SNVs were identified that were unique only to F1 (**Table 3**). These SNVs were located in the region from PA0717-PA0729 (**Figure 5A**) with the PseudoCAP class function of “related to phage, transposon and plasmid.” Three SNVs were from intergenic regions while 61 were from coding regions. Of the 61 SNVs, 18 were transversions while the rest was transitions. Furthermore, 11 of the 61 SNVs in the coding region, 11 SNVs correspond to non-synonymous mutations while the remainder 50 SNVs corresponds to synonymous mutations (**Figures 5B,C**). It was observed that transversions and non-synoymous SNVs tend to congregate nearer to the 5′ end of the region (**Figure 5B**).

**Table 3:** Single nucleotide variations unique to F1 strain compared to the wild-type.

Locus (No. SNVs)	Position	Ref base	Genotype call	Ref amino acid	Amino acid call	PseudoCAP class function	
PA0719 **(3)**	789941^a^	C	G	Ser	Arg	Hypothetical	Related to phage, transposon, or plasmid
	789960	A	C	Arg	Arg		
	789985^a^	C	A	Ser	Tyr		
PA0720 **(11)**	790228	T	C	Tyr	Tyr	DNA replication, recombination, modification and repair	Related to phage, transposon, or plasmid
	790267	A	G	Gln	Gln		
	790312	A	C	Gly	Gly		
	790360	C	T	Ile	Ile		
	790408^b^	T	C	Arg	Arg		
	790411^b^	G	C	Pro	Pro		
	790447^b^	G	A	Gln	Gln		
	790453^b^	A	T	Leu	Leu		
	790521^a,b^	T	C	Val	Ala		
	790540^b^	C	T	Arg	Arg		
	790580^a,b^	A	T	Thr	Ser		
Intergenic	790604^b^	C	G				
PA0724 **(4)**
	791486	G	A	Ser	Ser		Related to phage, transposon, or plasmid	
	791518^a^	C	A	Ala	Asp		
	791626^a^	G	C	Ala	Gly		
	791651	C	T	Gly	Gly		
PA0726 **(11)**	793023^b^	C	G	Pro	Pro	Hypothetical	Related to phage, transposon, or plasmid		
793026^b^	T	C	Asn	Asn		
793056	G	A	Gln	Gln		
793371	C	G	Leu	Leu		
793374	C	T	Asp	Asp		
793389	T	C	His	His		
793410	T	C	Ile	Ile		
793411^a^	G	A	Val	Ile		
793432^a^	T	G	Ser	Ala		
793437	C	T	Tyr	Tyr		
793449	T	C	Asp	Asp		
PA0727 **(19)**	794872	G	A	Glu	Glu	Hypothetical	Related to phage, transposon, or plasmid
	794875	A	G	Val	Val		
	795082	G	T	Arg	Arg		
	795289	C	G	Val	Val		
	795307	C	T	Arg	Arg		
	795331	G	A	Gln	Gln		
	795349	C	T	Arg	Arg		
	795355	T	C	Ala	Ala		
	795361	A	G	Gly	Gly		
	795391	G	C	Gly	Gly		
	795394	T	C	Leu	Leu		
	795403	C	T	His	His		
	795457	G	A	Thr	Thr		
	795499	C	T	Ala	Ala		
	795553	T	C	Tyr	Tyr		
	795574	T	C	Phe	Phe		
	795583	T	C	Arg	Arg		
	795598	T	C	Phe	Phe		
	795652	T	G	Ala	Ala		
PA0728 **(10)**	795867	G	A	Lys	Lys	Putative enzymes	Related to phage, transposon, or plasmid
796015	T	C	Leu	Leu		
796035	C	T	Cys	Cys		
796125	A	G	Arg	Arg		
796161	C	T	Asn	Asn		
796203	C	T	Arg	Arg		
796273	T	C	Leu	Leu		
796443	T	C	Phe	Phe		
796446	C	T	Ala	Ala		
	796452	T	C	Thr	Thr		
Intergenic	796789	T	G			
	797040	T	C			
PA0729 **(3)**	797516	T	C	Val	Ala	Hypothetical
	797533	T	C	Leu	Leu	
	797550	C	T	Ser	Ser	


*^a^Denotes a non-synonymous SNV. ^b^Denotes SNVs that are not found in the other 4 SCVs isolated.*

It is puzzling that F1 carried so many synonymous mutations that led to no change in amino acids in the coded proteins. Synonymous mutations may affect the regulation of protein expression at the stage of translation whereby certain codons are much preferred to code for a particular amino acid. This form of codon usage bias has been extensively described in *Escherichia coli* (Sharp et al., 1988; Pek et al., 2015). Studying codon bias in *P. aeruginosa*, Grocock and Sharp (2002) concluded that a weak codon bias is present in *P. aeruginosa* and calculated relative synonymous codon usage (RSCU) values for each codon. Using their RSCU values, we investigated whether there is a trend of F1 using preferred codons, as specified by its 50 synonymous mutations, to code for particular amino acids. Synonymous SNVs were split into two groups based on whether a less or more efficient codon for a particular amino acid was used. We identified 29 SNVs where a preferred codon was used and 22 SNVs where a less-preferred codon was used. For each of these groups, the proportion for each class of amino acid side chain that was found in the group was counted. We deduced that aromatic and BCAAs were preferentially coded using more efficient codons (**Figure 5D**). An overview of these findings is presented in **Figure 5D** which displays the type of SNV at each position. To address whether such SNVs are common among all SCVs after infecting *P. aeruginosa* with PB1 phage, we isolated four additional SCVs derived from PB1 infection of F0 and sequenced their genomes. 55 of the 61 SNVs that were identified in F1 were also present in these 4 SCVs (**Figure 5E**).

## Discussion

In this study, SCV were isolated from the surviving population of wild-type *P. aeruginosa* PAO1 after 24 h of exposure to PB1 phage. The SCV isolates were found to be resistant to further PB1 infection and displayed several phenotypic changes. Compared to the wild-type F0, the PB1 phage-resistant F1 are less competent at attaching to surfaces in the biofilm formation assay (**Figure 2A**), produces lower amounts of the virulence factors elastase and pyocyanin (**Figures 2B,C**), and do not twitch as much (**Figure 2D**). Since both attachment and twitching are important processes in the formation of biofilms (O’Toole and Kolter, 1998; Ramsey and Whiteley, 2004), F0 would be expected to have greater biofilm formation than F1. Consistent with reports on bacterial SCVs, reduced twitching ability is also associated with smaller colonies on agar (Proctor et al., 2006). The surface of F1 is also more hydrophobic than F0 (**Figure 2E**), allowing F1 cells to aggregate more easily through hydrophobic interactions and hence able to form smaller-sized colonies than F0, similar to clinical variants reported by Kirisits et al. (2005). The production of virulence factors, pyocyanin and elastase (**Figures 2D,E**) are co-regulated through the *las* quorum sensing system (Pearson et al., 1997; Glessner et al., 1999; Sauer et al., 2002). The lower expression of both factors in F1 may indicate that the *las* system is deficient in F1 strains.

From the microarray data, several genes from *P. aeruginosa* serotype O5 OSA biosynthesis cluster were found to be upregulated. OSA is made up of repeating units of sugars which acts as the serotype specific O antigen (Burrows et al., 1996). The structure of OSA varies among the different *P. aeruginosa* serotypes and PAO1 strain belongs to the O5 serotype. OSA has a varying chain length based on the number of repeating units (Rocchetta and Lam, 1997). The chain length of the OSA is regulated by *wzz* (Daniels et al., 2002). The expression pattern of several OSA genes was upregulated in the F1 over the F0 strain (**Table 2A**), indicating alteration of the structure of OSA in F1. These observations were supported through Western blot analysis of LPS preparations using antibodies specific to the inner, outer core and OSA. The results showed a markedly different distribution of OSA banding pattern between the two strains. We observed the absence of high molecular weight OSA and lower amounts of low molecular weight OSA in the F1 over the F0 strain. This could have contribute to the lower surface hydrophobicity observed in the MATH assay which suggest change in structure of LPS. In the study by Garbe et al. (2010), they concluded that the receptor for the PB1-like phage JG024 is a component of the core LPS. Changes to the OSA could play a role in preventing binding of phage particles and initializing the infection process. From these results, we can postulate that differences in surface properties between the strains may prevent PB1 phage from binding to LPS receptors on F1 surface.

Infection by PB1 phages could have induced a stress response in the *P. aeruginosa* that caused the bacteria to accumulate spontaneous mutations. Phage-driven changes in phenotype have been previously shown for PP7, a ssRNA, pilus-binding phage. Similar to PB1, PP7 induces SCVs in *P. aeruginosa* (Brockhurst et al., 2005). Under stressful conditions, the SOS response in bacteria is activated, thus allowing the bacteria to undergo mutations on a genome wide scale (McKenzie et al., 2000). Certain regions of the genome tend to be hypermutable, and indeed, our sequencing results show that SNVs were predominantly found in the Pf1 prophage region, located at positions PA0719–PA0729 of the *P. aeruginosa* genome. This region originated from a horizontal gene transfer event from Pf1 filamentous lysogenic phages, to ensure that their genetic material is preserved in the bacteria, and has an unusually low GC-content when compared to the rest of the genome. In a study by Wei et al. (2011), where *P. aeruginosa* SCV was generated by exposing wild-type bacteria to high concentrations of gentamicin. One key difference, however, is that the SCV in our study showed an increased susceptibility to ciprofloxacin, while theirs showed an increased resistance to the antibiotic. They also discovered a silent mutation in the elongation factor *tufA* in revertant cells that could be the cause of their increased growth rate. In a similar fashion, the silent mutations found in the Pf1 region in this study may contribute to codon bias and resulted in one or more of the phenotypes observed. Filamentous phage have been known to exhibit high frequency of mutations which could explain the presence of SNVs found in the Pf1 region (Kuo et al., 2000). Previous studies have shown that cells can acquire a superinfective phenotype, capable of killing cells in *P. aeruginosa* biofilms, through mutations in the Pf1 region, (Webb et al., 2003). Similarly, Pf1 prophage might be involved in the selection of F1 when F0 was first exposed to PB1 phage. This implicates a possible role in the Pf1 prophage region of *P. aeruginosa* for the generation of the SCV phenotype.

## Author Contributions

WL designed and performed the experiments, contributed to the results interpretation, manuscript writing and approval of the final version for publication; KP performed the experiments, contributed to the results interpretation and approval of the final version for publications; AT, SL and DO contributed to the results interpretation and approval of the final version for publication.

## Conflict of Interest Statement

The authors declare that the research was conducted in the absence of any commercial or financial relationships that could be construed as a potential conflict of interest.

## Footnotes

1 usegalaxy.org

2 www.pseudomonas.com

## References

[B1] Brockhurst M. A. Buckling A. Rainey P. B. (2005). The effect of a bacteriophage on diversification of the opportunistic bacterial pathogen, *Pseudomonas aeruginosa*. *Proc. R. Soc. Lon. B Biol. Sci.* 272 1385–1391. 10.1098/rspb.2005.3086 PMC1560335 16006335

[B2] Burrows L. Charter D. Lam J. (1996). Molecular characterization of the *Pseudomonas aeruginosa* serotype O5 (PAO1) B-band lipopolysaccharide gene cluster. *Mol. Microbiol.* 22 481–495. 10.1046/j.1365-2958.1996.1351503.x 8939432

[B3] Carlsson M. Shukla S. Petersson A. C. Segelmark M. Hellmark T. (2011). *Pseudomonas aeruginosa* in cystic fibrosis: pyocyanin negative strains are associated with BPI-ANCA and progressive lung disease. *J. Cystic Fibros.* 10 265–271. 10.1016/j.jcf.2011.03.004 21463973

[B4] Ceyssens P. J. Miroshnikov K. Mattheus W. Krylov V. Robben J. Noben J. P. (2009). Comparative analysis of the widespread and conserved PB1-like viruses infecting *Pseudomonas aeruginosa*. *Environ. Microbiol.* 11 2874–2883. 10.1111/j.1462-2920.2009.02030.x 19678828

[B5] Daniels C. Griffiths C. Cowles B. Lam J. S. (2002). *Pseudomonas aeruginosa* O-antigen chain length is determined before ligation to lipid A core. *Environ. Microbiol.* 4 883–897. 10.1046/j.1462-2920.2002.00288.x 12534470

[B6] Davis M. R. Jr. Goldberg J. B. (2012). Purification and visualization of lipopolysaccharide from Gram-negative bacteria by hot aqueous-phenol extraction. *J. Vis. Exp.* 63 e3916 10.3791/3916 PMC3466933 22688346

[B7] DiMasi J. A. Hansen R. W. Grabowski H. G. (2003). The price of innovation: new estimates of drug development costs. *J. Health Econ.* 22 151–185. 10.1016/S0167-6296(02)00126-1 12606142

[B8] Garbe J. Wesche A. Bunk B. Kazmierczak M. Selezska K. Rohde C. (2010). Characterization of JG024, a *Pseudomonas aeruginosa* PB1-like broad host range phage under simulated infection conditions. *BMC Microbiol.* 10:301 10.1186/1471-2180-10-301 PMC3008698 21110836

[B9] Gilbert J. Henske P. Singh A. (2003). Rebuilding big pharma’s business model. *In Vivo* 21 73–80.

[B10] Glessner A. Smith R. S. Iglewski B. H. Robinson J. B. (1999). Roles of *Pseudomonas aeruginosa* las and rhl quorum-sensing systems in control of twitching motility. *J. Bacteriol.* 181 1623–1629. 10049396 10.1128/jb.181.5.1623-1629.1999 PMC93554

[B11] Govan J. Deretic V. (1996). Microbial pathogenesis in cystic fibrosis: mucoid *Pseudomonas aeruginosa* and *Burkholderia cepacia*. *Microbiol. Rev.* 60 539–574. 8840786 10.1128/mr.60.3.539-574.1996 PMC239456

[B12] Grocock R. J. Sharp P. M. (2002). Synonymous codon usage in *Pseudomonas aeruginosa* PA01. *Gene* 289 131–139. 10.1016/S0378-1119(02)00503-6 12036591

[B13] Hackstadt A. J. Hess A. M. (2009). Filtering for increased power for microarray data analysis. *BMC Bioinformatics* 10:11 10.1186/1471-2105-10-11 PMC2661050 19133141

[B14] Harper D. R. Enright M. C. (2011). Bacteriophages for the treatment of *Pseudomonas aeruginosa* infections. *J. Appl. Microbiol.* 111 1–7. 10.1111/j.1365-2672.2011.05003.x 21410851

[B15] Holloway B. W. Egan J. B. Monk M. (1960). Lysogeny in *Pseudomonas aeruginosa*[ast]. *Aust. J. Exp. Biol. Med.* 38 321–330. 10.1038/icb.1960.34 13715401

[B16] Hui J. G. Mai-Prochnow A. Kjelleberg S. McDougald D. Rice S. A. (2014). Environmental cues and genes involved in establishment of the superinfective Pf4 phage of *Pseudomonas aeruginosa*. *Front. Microbiol.* 5:654 10.3389/fmicb.2014.00654 PMC4251444 25520708

[B17] Kamath S. Kapatral V. Chakrabarty A. (1998). Cellular function of elastase in *Pseudomonas aeruginosa*: role in the cleavage of nucleoside diphosphate kinase and in alginate synthesis. *Mol. Microbiol.* 30 933–941. 10.1046/j.1365-2958.1998.01121.x 9988471

[B18] Keen E. C. (2012). Phage therapy: concept to cure. *Front. Microbiol.* 3:238 10.3389/fmicb.2012.00238 PMC3400130 22833738

[B19] Khawaldeh A. Morales S. Dillon B. Alavidze Z. Ginn A. N. Thomas L. (2011). Bacteriophage therapy for refractory *Pseudomonas aeruginosa* urinary tract infection. *J. Med. Microbiol.* 60 1697–1700. 10.1099/jmm.0.029744-0 21737541

[B20] King E. O. Ward M. K. Raney D. E. (1954). Two simple media for the demonstration of pyocyanin and fluorescin. *J. Lab. Clin. Med.* 44 301–307. 13184240

[B21] Kirisits M. J. Prost L. Starkey M. Parsek M. R. (2005). Characterization of colony morphology variants isolated from *Pseudomonas aeruginosa* biofilms. *Appl. Environ. Microbiol.* 71 4809–4821. 10.1128/AEM.71.8.4809-4821.2005 16085879 PMC1183349

[B22] Kirschke D. L. Jones T. F. Craig A. S. Chu P. S. Mayernick G. G. Patel J. A. (2003). *Pseudomonas aeruginosa* and *Serratia marcescens* contamination associated with a manufacturing defect in bronchoscopes. *N. Engl. J. Med.* 348 214–220. 10.1056/NEJMoa021791 12529461

[B23] Kropinski A. M. Chan L. Jarrell K. Milazzo F. (1977). The nature of *Pseudomonas aeruginosa* strain PAO bacteriophage receptors. *Can. J. Microbiol.* 23 653–658. 10.1139/m77-098 406024

[B24] Krylov V. Shaburova O. Krylov S. Pleteneva E. (2013). A genetic approach to the development of new therapeutic phages to fight *Pseudomonas aeruginosa* in wound infections. *Viruses* 5 15–53. 10.3390/v5010015 23344559 PMC3564109

[B25] Krylov V. Tolmachova T. Akhverdian V. (1993). DNA homology in species of bacteriophages active on *Pseudomonas aeruginosa*. *Arch. Virol.* 131 141–151. 10.1007/BF01379086 8328909

[B26] Kuo M.-Y. Yang M.-K. Chen W.-P. Kuo T.-T. (2000). High-frequency interconversion of turbid and clear plaque strains of bacteriophage f1 and associated host cell death. *Can. J. Microbiol.* 46 841–847. 10.1139/w00-068 11006845

[B27] Kwan T. Liu J. DuBow M. Gros P. Pelletier J. (2006). Comparative genomic analysis of 18 *Pseudomonas aeruginosa* bacteriophages. *J. Bacteriol.* 188 1184–1187. 10.1128/JB.188.3.1184-1187.2006 16428425 PMC1347338

[B28] Lang A. B. Horn M. P. Imboden M. A. Zuercher A. W. (2004). Prophylaxis and therapy of *Pseudomonas aeruginosa* infection in cystic fibrosis and immunocompromised patients. *Vaccine* 22 S44–S48. 10.1016/j.vaccine.2004.08.016 15576201

[B29] Lau G. W. Hassett D. J. Ran H. Kong F. (2004). The role of pyocyanin in *Pseudomonas aeruginosa* infection. *Trends Mol. Med.* 10 599–606. 10.1016/j.molmed.2004.10.002 15567330

[B30] Ly-Chatain M. H. (2014). The factors affecting effectiveness of treatment in phages therapy, mini review. *Front. Microbiol.* 5:51 10.3389/fmicb.2014.00051 PMC3927074 24600439

[B31] Lyczak J. B. Cannon C. L. Pier G. B. (2000). Establishment of *Pseudomonas aeruginosa* infection: lessons from a versatile opportunist. *Microbes Infect.* 2 1051–1060. 10.1016/S1286-4579(00)01259-4 10967285

[B32] McKenzie G. J. Harris R. S. Lee P. L. Rosenberg S. M. (2000). The SOS response regulates adaptive mutation. *Proc. Natl. Acad. Sci. U.S.A.* 97 6646–6651. 10.1073/pnas.120161797 10829077 PMC18688

[B33] McVay C. S. Velásquez M. Fralick J. A. (2007). Phage therapy of *Pseudomonas aeruginosa* infection in a mouse burn wound model. *Antimicrob. Agents Chemother.* 51 1934–1938. 10.1128/AAC.01028-06 17387151 PMC1891379

[B34] Merabishvili M. Pirnay J.-P. Verbeken G. Chanishvili N. Tediashvili M. Lashkhi N. (2009). Quality-controlled small-scale production of a well-defined bacteriophage cocktail for use in human clinical trials. *PLoS ONE* 4:e4944 10.1371/journal.pone.0004944 PMC2654153 19300511

[B35] Morello E. Saussereau E. Maura D. Huerre M. Touqui L. Debarbieux L. (2011). Pulmonary bacteriophage therapy on *Pseudomonas aeruginosa* cystic fibrosis strains: first steps towards treatment and prevention. *PLoS ONE* 6:e16963 10.1371/journal.pone.0016963 PMC3039662 21347240

[B36] Nickel J. Ruseska I. Wright J. Costerton J. (1985). Tobramycin resistance of *Pseudomonas aeruginosa* cells growing as a biofilm on urinary catheter material. *Antimicrob. Agents Chemother.* 27 619–624. 10.1128/AAC.27.4.619 3923925 PMC180108

[B37] O’Toole G. A. Kolter R. (1998). Flagellar and twitching motility are necessary for *Pseudomonas aeruginosa* biofilm development. *Mol. Microbiol.* 30 295–304. 10.1046/j.1365-2958.1998.01062.x 9791175

[B38] Pearson J. P. Pesci E. C. Iglewski B. H. (1997). Roles of *Pseudomonas aeruginosa* las and rhl quorum-sensing systems in control of elastase and rhamnolipid biosynthesis genes. *J. Bacteriol.* 179 5756–5767. 9294432 10.1128/jb.179.18.5756-5767.1997 PMC179464

[B39] Pek H. B. Klement M. Ang K. S. Chung B. K.-S. Ow D. S.-W. Lee D.-Y. (2015). Exploring codon context bias for synthetic gene design of a thermostable invertase in *Escherichia coli*. *Enzyme Microb. Technol.* 75 57–63. 10.1016/j.enzmictec.2015.04.008 26047917

[B40] Pérez P. F. Minnaard Y. Disalvo E. A. De Antoni G. L. (1998). Surface properties of bifidobacterial strains of human origin. *Appl. Environ. Microbiol.* 64 21–26. 9435057 10.1128/aem.64.1.21-26.1998 PMC124666

[B41] Pleteneva E. Shaburova O. Sykilinda N. Miroshnikov K. Kadykov V. Krylov S. (2008). Study of the diversity in a group of phages of *Pseudomonas aeruginosa* species PB1 (Myoviridae) and their behavior in adsorbtion-resistant bacterial mutants. *Russ. J. Genet.* 44 150–158. 10.1134/S1022795408020051 18619036

[B42] Poole K. (2004). Efflux-mediated multiresistance in Gram-negative bacteria. *Clin. Microbiol. Infect.* 10 12–26. 10.1111/j.1469-0691.2004.00763.x 14706082

[B43] Proctor R. A. Von Eiff C. Kahl B. C. Becker K. McNamara P. Herrmann M. (2006). Small colony variants: a pathogenic form of bacteria that facilitates persistent and recurrent infections. *Nat. Rev. Microbiol.* 4 295–305. 10.1038/nrmicro1384 16541137

[B44] Ramsey M. M. Whiteley M. (2004). *Pseudomonas aeruginosa* attachment and biofilm development in dynamic environments. *Mol. Microbiol.* 53 1075–1087. 10.1111/j.1365-2958.2004.04181.x 15306012

[B45] Rashid M. H. Kornberg A. (2000). Inorganic polyphosphate is needed for swimming, swarming, and twitching motilities of *Pseudomonas aeruginosa*. *Proc. Natl. Acad. Sci. U.S.A.* 97 4885–4890. 10.1073/pnas.060030097 10758151 PMC18327

[B46] Rocchetta H. L. Lam J. S. (1997). Identification and functional characterization of an ABC transport system involved in polysaccharide export of A-band lipopolysaccharide in *Pseudomonas aeruginosa*. *J. Bacteriol.* 179 4713–4724. 9244257 10.1128/jb.179.15.4713-4724.1997 PMC179316

[B47] Sauer K. Camper A. K. Ehrlich G. D. Costerton J. W. Davies D. G. (2002). *Pseudomonas aeruginosa* displays multiple phenotypes during development as a biofilm. *J. Bacteriol.* 184 1140–1154. 10.1128/jb.184.4.1140-1154.2002 11807075 PMC134825

[B48] Sharp P. M. Cowe E. Higgins D. G. Shields D. C. Wolfe K. H. Wright F. (1988). Codon usage patterns in *Escherichia coli*, *Bacillus subtilis*, *Saccharomyces cerevisiae*, *Schizosaccharomyces pombe*, *Drosophila melanogaster* and *Homo sapiens*; a review of the considerable within-species diversity. *Nucleic Acids Res.* 16 8207–8211. 10.1093/nar/16.17.8207 3138659 PMC338553

[B49] Spellberg B. Powers J. H. Brass E. P. Miller L. G. Edwards J. E. (2004). Trends in antimicrobial drug development: implications for the future. *Clin. Infect. Dis.* 38 1279–1286. 10.1086/420937 15127341

[B50] Stamm W. E. (1978). Infections related to medical devices. *Ann. Int. Med.* 89 764–769. 10.7326/0003-4819-89-5-764 717950

[B51] Stepanović S. Vuković D. Dakić I. Savić B. Švabić-Vlahović M. (2000). A modified microtiter-plate test for quantification of staphylococcal biofilm formation. *J. Microbiol. Methods* 40 175–179. 10.1016/S0167-7012(00)00122-6 10699673

[B52] Stover C. Pham X. Erwin A. Mizoguchi S. Warrener P. Hickey M. (2000). Complete genome sequence of *Pseudomonas aeruginosa* PAO1, an opportunistic pathogen. *Nature* 406 959–964. 10.1038/35023079 10984043

[B53] Sutton S. (2011). Measurement of microbial cells by optical density. *J. Validation Techn* 17 47–49.

[B54] System N. N. I. S. (2004). National nosocomial infections surveillance (NNIS) system report, data summary from January 1992 through June 2004, issued October 2004. *Am. J. Infect. Control* 32 470–485. 10.1016/j.ajic.2004.10.001 15573054

[B55] Talbot G. H. Bradley J. Edwards J. E. Gilbert D. Scheld M. Bartlett J. G. (2006). Bad bugs need drugs: an update on the development pipeline from the antimicrobial availability task force of the infectious diseases society of America. *Clin. Infect. Dis.* 42 657–668. 10.1086/499819 16447111

[B56] Taneja N. Emmanuel R. Chari P. Sharma M. (2004). A prospective study of hospital-acquired infections in burn patients at a tertiary care referral centre in North India. *Burns* 30 665–669. 10.1016/j.burns.2004.02.011 15475139

[B57] Watanabe R. Matsumoto T. Sano G. Ishii Y. Tateda K. Sumiyama Y. (2007). Efficacy of bacteriophage therapy against gut-derived sepsis caused by *Pseudomonas aeruginosa* in mice. *Antimicrob. Agents Chemother.* 51 446–452. 10.1128/AAC.00635-06 17116686 PMC1797723

[B58] Webb J. S. Lau M. Kjelleberg S. (2004). Bacteriophage and phenotypic variation in *Pseudomonas aeruginosa* biofilm development. *J. Bacteriol.* 186 8066–8073. 10.1128/JB.186.23.8066-8073.2004 15547279 PMC529096

[B59] Webb J. S. Thompson L. S. James S. Charlton T. Tolker-Nielsen T. Koch B. (2003). Cell death in *Pseudomonas aeruginosa* biofilm development. *J. Bacteriol.* 185 4585–4592. 10.1128/JB.185.15.4585-4592.2003 12867469 PMC165772

[B60] Wei Q. Tarighi S. Dötsch A. Häussler S. Müsken M. Wright V. J. (2011). Phenotypic and genome-wide analysis of an antibiotic-resistant small colony variant (SCV) of *Pseudomonas aeruginosa*. *PLoS ONE* 6:e29276 10.1371/journal.pone.0029276 PMC3240657 22195037

[B61] Wright A. Hawkins C. Änggård E. Harper D. (2009). A controlled clinical trial of a therapeutic bacteriophage preparation in chronic otitis due to antibiotic-resistant *Pseudomonas aeruginosa*; a preliminary report of efficacy. *Clin. Otolaryngol.* 34 349–357. 10.1111/j.1749-4486.2009.01973.x 19673983

[B62] Yoshimura F. Nikaido H. (1982). Permeability of *Pseudomonas aeruginosa* outer membrane to hydrophilic solutes. *J. Bacteriol.* 152 636–642. 6813310 10.1128/jb.152.2.636-642.1982 PMC221510

