# Supplementary material for: Small Colony Variants and Single Nucleotide Variations in Pf1 Region of PB1 Phage-Resistant Pseudomonas aeruginosa
Source: Front Microbiol. 2016 Mar 9;7:282. doi: 10.3389/fmicb.2016.00282 (PMC4783410; doi:10.3389/fmicb.2016.00282)
Supplement: Supplementary file 1 [file Table_1.PDF]

Table S1- Full list of up-regulated genes, arranged according to their fold changes.

Locus	*Gene Symbol*	Gene	p-value	Fold Change
PA1340	---	amino acid ABC transporter membrane protein	2.E-02	3.1
PA1339	---	amino acid ABC transporter ATP binding protein	5.E-03	2.9
PA1341	---	amino acid ABC transporter membrane protein	2.E-02	2.8
PA0527	*dnr*	transcriptional regulator Dnr	7.E-03	2.4
PA0080	---	---	2.E-03	2.4
PA3151	*hisF2*	imidazoleglycerol-phosphate synthase, cyclase subunit	1.E-02	2.4
PA5568	---	conserved hypothetical protein	8.E-03	2.3
PA3152	*hisH2*	glutamine amidotransferase	3.E-03	2.3
PA3149	*wbpH*	probable glycosyltransferase WbpH	3.E-02	2.2
PA1555	---	Cytochrome c oxidase, cbb3-type, CcoP subunit	2.E-02	2.2
PA3150	*wbpG*	LPS biosynthesis protein WbpG	2.E-02	2.1
PA5494	---	---	4.E-02	2.1
PA0729	---	---	2.E-03	1.9
PA0456	---	probable cold-shock protein	1.E-02	1.9
PA1421	*gbuA*	guanidinobutyrase	2.E-04	1.8
PA3148	*wbpI*	probable UDP-N-acetylglucosamine 2-epimerase WbpI	4.E-02	1.8
PA3645	*fabZ*	(3R)-hydroxymyristoyl-[acyl carrier protein] dehydratase	2.E-03	1.8
PA4272	*rplJ*	50S ribosomal protein L10	3.E-02	1.8
PA0086	---	---	3.E-02	1.8
PA5001	---	---	2.E-03	1.8
PA0408	*pilG*	twitching motility protein PilG	2.E-02	1.8
PA0082	---	---	3.E-05	1.7
PA5232	---	conserved hypothetical protein	4.E-02	1.7
PA0723	*coaB*	coat protein B of bacteriophage Pf1)	2.E-02	1.7
PA0079	---	---	6.E-04	1.7
PA4727	*pcnB*	poly(A) polymerase	8.E-03	1.7
PA0088	---	---	3.E-02	1.7
PA0563	---	---	5.E-03	1.7

PA3982	---	---	2.E-02	1.7
PA4348	---	---	4.E-03	1.7
PA5340	---	---	3.E-02	1.7
PA4321	---	---	5.E-02	1.7
PA0087	---	---	4.E-02	1.7
PA0094	---	---	3.E-02	1.7
PA0084	---	---	4.E-03	1.7
PA0610	*prtN*	transcriptional regulator PrtN	3.E-02	1.6
PA1155	*nrdB*	ribonucleoside reductase, small chain	1.E-02	1.6
PA0090	---	---	1.E-02	1.6
PA0078	---	---	1.E-04	1.6
PA4466	---	probable phosphoryl carrier protein	5.E-03	1.6
PA0083	---	---	2.E-02	1.6
PA0436	---	probable transcriptional regulator	2.E-02	1.6
PA4274	*rplK*	50S ribosomal protein L11	1.E-02	1.6
PA2459	---	---	7.E-03	1.6
PA0071	---		8.E-03	1.6
PA3156	*wbpD*	probable acetyltransferase WbpD	3.E-02	1.6
PA5492	---	---	1.E-02	1.6
PA1013	*purC*	phosphoribosylaminoimidazole-succinocarboxamide synthase	2.E-02	1.6
PA5329	---	conserved hypothetical protein	1.E-02	1.5
PA5244	---	conserved hypothetical protein	2.E-02	1.5
PA0362	*fdx1*	ferredoxin [4Fe-4S]	4.E-02	1.5
PA5414	---	---	2.E-02	1.5
PA4268	*rpsL*	30S ribosomal protein S12	5.E-02	1.5
PA0972	*tolB*	TolB protein	2.E-02	1.5
PA0722	---	hypothetical protein of bacteriophage Pf1	6.E-03	1.5
