# Supplementary material for: Small Colony Variants and Single Nucleotide Variations in Pf1 Region of PB1 Phage-Resistant Pseudomonas aeruginosa
Source: Front Microbiol. 2016 Mar 9;7:282. doi: 10.3389/fmicb.2016.00282 (PMC4783410; doi:10.3389/fmicb.2016.00282)
Supplement: Supplementary file 2 [file Table_2.PDF]

Table S2- Full list of down-regulated genes, arranged according to their fold changes.

Locus	*Gene Symbol*	Gene	p-value	Fold Change
PA5082	---	Transport of small molecules	3.E-02	-16.0
PA5083	---	---	3.E-02	-11.4
PA2204	---	probable binding protein component of ABC transporter	2.E-03	-7.3
PA2553	---	probable acyl-CoA thiolase	3.E-03	-5.6
PA1984	---	NAD+ dependent aldehyde dehydrogenase	9.E-04	-5.5
PA0745	---	probable enoyl-CoA hydratase/isomerase	1.E-03	-5.0
PA3570	*mmsA*	methylmalonate-semialdehyde dehydrogenase	6.E-03	-4.5
PA2114	---	probable major facilitator superfamily (MFS) transporter	3.E-03	-4.4
PA0049	---	---	6.E-05	-4.4
PA3569	*mmsB*	3-hydroxyisobutyrate dehydrogenase	7.E-04	-4.3
PA0744	---	probable enoyl-CoA hydratase/isomerase	1.E-03	-4.1
PA0865	*hpd*	4-hydroxyphenylpyruvate dioxygenase	8.E-03	-4.1
PA2113	---	pyroglutamate porin	2.E-03	-3.9
PA0794	---	probable aconitate hydratase	3.E-02	-3.8
PA2552	---	probable acyl-CoA dehydrogenase	1.E-03	-3.6
PA4023	---	probable transport protein	9.E-03	-3.5
PA3931	---	conserved hypothetical protein	7.E-03	-3.5
PA2112	---	---	8.E-03	-3.5
PA2249	*bkdB*	branched-chain alpha-keto acid dehydrogenase (lipoamide component)	1.E-03	-3.4
PA2248	*bkdA2*	2-oxoisovalerate dehydrogenase (beta subunit)	4.E-03	-3.4
PA3186	*oprB*	Glucose/carbohydrate outer membrane porin OprB precursor	2.E-03	-3.3
PA2008	*fahA*	fumarylacetoacetase	5.E-03	-3.2
PA4502	---	probable binding protein component of ABC transporter	1.E-02	-3.2
PA4024	*eutB*	ethanolamine ammonia-lyase large subunit	2.E-02	-3.2
PA0283	*sbp*	sulfate-binding protein precursor	1.E-02	-3.1
PA2250	*lpdV*	lipoamide dehydrogenase-Val	4.E-03	-3.0
PA0746	---	probable acyl-CoA dehydrogenase	3.E-03	-3.0

PA0447	*gcdH*	glutaryl-CoA dehydrogenase	7.E-04	-3.0
PA2111	---	---	5.E-03	-2.9
PA3187	---	probable ATP-binding component of ABC transporter	2.E-03	-2.9
PA4496	---	probable binding protein component of ABC transporter	1.E-02	-2.9
PA4505	---	probable ATP-binding component of ABC transporter	5.E-03	-2.8
PA0284	---	---	6.E-03	-2.8
PA0866	*aroP2*	aromatic amino acid transport protein AroP2	6.E-04	-2.8
PA4504	---	probable permease of ABC transporter	1.E-02	-2.8
PA4506	---	probable ATP-binding component of ABC dipeptide transporter	2.E-04	-2.8
PA0793	---	---	5.E-02	-2.7
PA4503	---	probable permease of ABC transporter	2.E-02	-2.7
PA2247	*bkdA1*	2-oxoisovalerate dehydrogenase (alpha subunit)	1.E-02	-2.7
PA3049	*rmf*	ribosome modulation factor	2.E-02	-2.7
PA2009	*hmgA*	homogentisate 1,2-dioxygenase	2.E-02	-2.7
PA2007	*maiA*	maleylacetoacetate isomerase	3.E-02	-2.7
PA3188	---	probable permease of ABC sugar transporter	4.E-03	-2.6
PA2013	*gnyH*	gamma-carboxygeranoyl-CoA hydratase, GnyH	2.E-02	-2.6
PA2012	*gnyA*	alpha subunit of geranoyl-CoA carboxylase, GnyA	4.E-02	-2.6
PA5415	*glyA1 /// glyA2*	serine hydroxymethyltransferase /// serine hydroxymethyltransferase	2.E-03	-2.5
PA0792	*prpD*	propionate catabolic protein PrpD	2.E-02	-2.5
PA3779	---	---	2.E-02	-2.5
PA1837	---	---	2.E-03	-2.5
PA3190	---	probable binding protein component of ABC sugar transporter	2.E-02	-2.5
PA5348	---	probable DNA-binding protein	2.E-02	-2.4
PA2445	*gcvP2*	glycine cleavage system protein P2	9.E-03	-2.3
PA2014	*gnyB*	beta subunit of geranoyl-CoA carboxylase, GnyB	2.E-02	-2.3
PA1838	*cysI*	sulfite reductase	3.E-02	-2.3
PA4443	*cysD*	ATP sulfurylase small subunit	2.E-02	-2.2
PA4607	---	---	9.E-04	-2.2
PA2443	*sdaA*	L-serine dehydratase	1.E-02	-2.2

PA3195	*gapA*	glyceraldehyde 3-phosphate dehydrogenase	5.E-03	-2.2
PA0870	*phhC*	aromatic amino acid aminotransferase	1.E-02	-2.2
PA3189	---	probable permease of ABC sugar transporter	4.E-02	-2.2
PA2554	---	probable short-chain dehydrogenase	1.E-02	-2.2
PA5100	*hutU*	urocanase	6.E-03	-2.1
PA1818	---	lysine-specific pyridoxal 5'-phosphate-dependent carboxylase	1.E-02	-2.1
PA5153	---	amino acid (lysine/arginine/ornithine/histidine/octopine) ABC transporter periplasmic binding protein	5.E-02	-2.1
PA0446	---	---	6.E-04	-2.0
PA3182	*pgl*	6-phosphogluconolactonase	1.E-02	-2.0
PA2001	*atoB*	acetyl-CoA acetyltransferase	1.E-02	-1.9
PA0897	*aruG*	arginine/ornithine succinyltransferase AII subunit	3.E-03	-1.9
PA0871	*phhB*	pterin-4-alpha-carbinolamine dehydratase	3.E-02	-1.9
PA2444	*glyA2*	serine hydroxymethyltransferase	2.E-02	-1.8
PA2128	*cupA1*	fimbrial subunit CupA1	7.E-03	-1.8
PA3181	---	2-keto-3-deoxy-6-phosphogluconate aldolase	2.E-02	-1.8
PA3068	*gdhB*	NAD-dependent glutamate dehydrogenase	2.E-02	-1.8
PA2634	---	isocitrate lyase	4.E-02	-1.8
PA4500	---	probable binding protein component of ABC transporter	4.E-02	-1.8
PA2850	*ohr*	organic hydroperoxide resistance protein	2.E-02	-1.8
PA0050	---	---	2.E-02	-1.8
PA1288	---	probable outer membrane protein precursor	3.E-02	-1.7
PA1537	---	probable short-chain dehydrogenase	4.E-02	-1.7
PA2442	*gcvT2*	glycine cleavage system protein T2	1.E-02	-1.6
PA1074	*braC*	branched-chain amino acid transport protein BraC	1.E-03	-1.6
PA5545	---	---	5.E-02	-1.6
PA1978	---	glycerol metabolism activator	2.E-04	-1.6
PA3183	*zwf*	glucose-6-phosphate 1-dehydrogenase	2.E-02	-1.6
PA3836	---	---	1.E-02	-1.6
PA0913	*mgtE*	probable Mg transporter MgtE	4.E-02	-1.6
PA0747	---	probable aldehyde dehydrogenase	4.E-02	-1.6
PA3712	---	---	8.E-03	-1.6

PA2000	---	dehydrocarnitine CoA transferase, subunit B	5.E-02	-1.6
PA1071	*braF*	branched-chain amino acid transport protein BraF	3.E-02	-1.6
PA4025	---	probable ethanolamine ammonia-lyase light chain	3.E-02	-1.5
PA2062	---	probable pyridoxal-phosphate dependent enzyme	4.E-02	-1.5
PA0899	*aruB*	succinylarginine dihydrolase	6.E-03	-1.5
